# Regenerative medicine and war: a front-line focus for UK defence

**DOI:** 10.1038/s41536-018-0053-4

**Published:** 2018-08-21

**Authors:** Abigail M. Spear, Graham Lawton, Robert M. T. Staruch, Rory F. Rickard

**Affiliations:** 1 Defence Science & Technology Laboratory, Porton Down, Salisbury, UK; 2 Academic Department of Military Surgery & Trauma, Royal Centre for Defence Medicine, Birmingham, UK

## Abstract

The recent prolonged conflicts in Iraq and Afghanistan saw the advancement of deployed trauma care to a point never before seen in war. The rapid translation of lessons from combat casualty care research, facilitated by an appetite for risk, contributed to year-on-year improvements in care of the injured. These paradigms, however, can only ever halt the progression of damage. Regenerative medicine approaches, in contrast, hold a truly disruptive potential to go beyond the cessation of damage from blast or ballistic trauma, to stimulate its reversal, and to do so from a very early point following injury. The internationally distributed and, in parts austere environments in which operational medical care is delivered provide an almost unique challenge to the development and translation of regenerative medicine technologies. In parallel, however, an inherent appetite for risk means that Defence will always be an early adopter. In focusing our operational priorities for regenerative medicine, the authors conducted a review of the current research landscape in the UK and abroad and sought wide clinical opinion. Our priorities are all applicable very far forward in the patient care pathway, and are focused on three broad and currently under-researched areas, namely: (a) blood, as an engineered tissue; (b) the mechanobiology of deep tissue loss and mechanobiological approaches to regeneration, and; (c) modification of the endogenous response. In focusing on these areas, we hope to engender the development of regenerative solutions for improved functional recovery from injuries sustained in conflict.

## Introduction

Conceptually, the risk of death or permanent disability during war has two components: (a) the prior risk of injury, *prius periculo*, linked to the relative offensive capability of one’s opponent, one’s own defensive capability, and the environment in which the war is conducted, and; (b) the post hoc capability of each force to save life and reduce disability following injury. The recent conflicts in Iraq and Afghanistan saw the development of allied post hoc trauma care to a point of capability never before achieved in war. The rapid delivery of resource-rich medical care close to the point of wounding, supported by a comprehensive and reactive logistics chain led to year-on-year improvements in survival rates from devastating injuries. Towards the end of those conflicts in 2014, British Service personnel were likely to survive injuries almost twice as severe as were survivable in 2003.^1^

Beyond the early, primary management of these casualties, however, were significant medical and logistical challenges. Following initial resuscitation, a secondary injury, exacerbated in some cases by sepsis, frequently caused progressive tissue death over days and weeks, often resulted in multiorgan failure, and occasionally resulted in death. In operations in Afghanistan, Britain lost 453 servicemen and women. A further 2209 were injured.^2^ Of those who died in medical hands, three tenths died in critical care units in the UK.^3^ As of 26 April 2017, in Afghanistan, allies in the United States of America had suffered 2346 deaths and 20,092 wounded in action.^4^ In survivors of that conflict, the magnitude of primary and secondary tissue loss demanded novel reconstructive approaches and comprehensive programmes of functional rehabilitation.^5^

Future British combat operations may not be carried out in such a small geographical area as Helmand Province. This, together with potentially unassured air superiority, would result in longer casualty evacuation timelines, possibly by land or sea. Casualties may not be able to access such a highly developed medical chain. Survival rates may initially falter, but it is in the area of secondary injury where prolonged evacuation timelines create most risk to our people.

Improvements in post hoc trauma care during the last 15 years of conflict were driven in part by the rapid delivery of lessons from research into targeted interventions to arrest primary injury and to mitigate secondary injury. Further incremental gains within this research area are likely. These paradigms, however, can only ever halt the progression of damage. In contrast, regenerative medicine has a truly disruptive potential, to go beyond the cessation of deterioration to stimulate reversal of this damage, and to do so from a very early point following injury. The field therefore holds a good deal of promise in the management of severe traumatic injury resulting from combat operations. Research in this area, however, has been relatively sparse and UK Defence’s engagement to date could be judged as reactionary and ad hoc.

The scoping study presented here, carried out jointly by the Defence, Science and Technology Laboratory (Dstl) and the Academic Department of Military Surgery and Trauma at the Royal Centre for Defence Medicine (RCDM) has defined our operational research priorities within regenerative medicine. Our methodology combined an assessment of clinical requirements with a review of the current regenerative medicine research landscape in the UK and internationally, while being cognisant of research programmes currently driven by allies. This approach has directed us to focus on traumatic injury as a result of conflict. Traumatic injuries are, of course, not confined to a military population. Combat trauma does, however, have some unique facets that provide the context for, and reasoning behind, the research priorities we subsequently lay out.

## The unique nature of combat trauma

Traumatic injuries, whether military or civilian, are a leading cause of mortality and morbidity worldwide, accounting for around 10% of the global burden of disease.^6,7^ Worldwide, 16,000 people succumb to injuries every day,^8^ and for every trauma death there are two survivors with serious and debilitating injuries.^9^

**Fig. 1:**
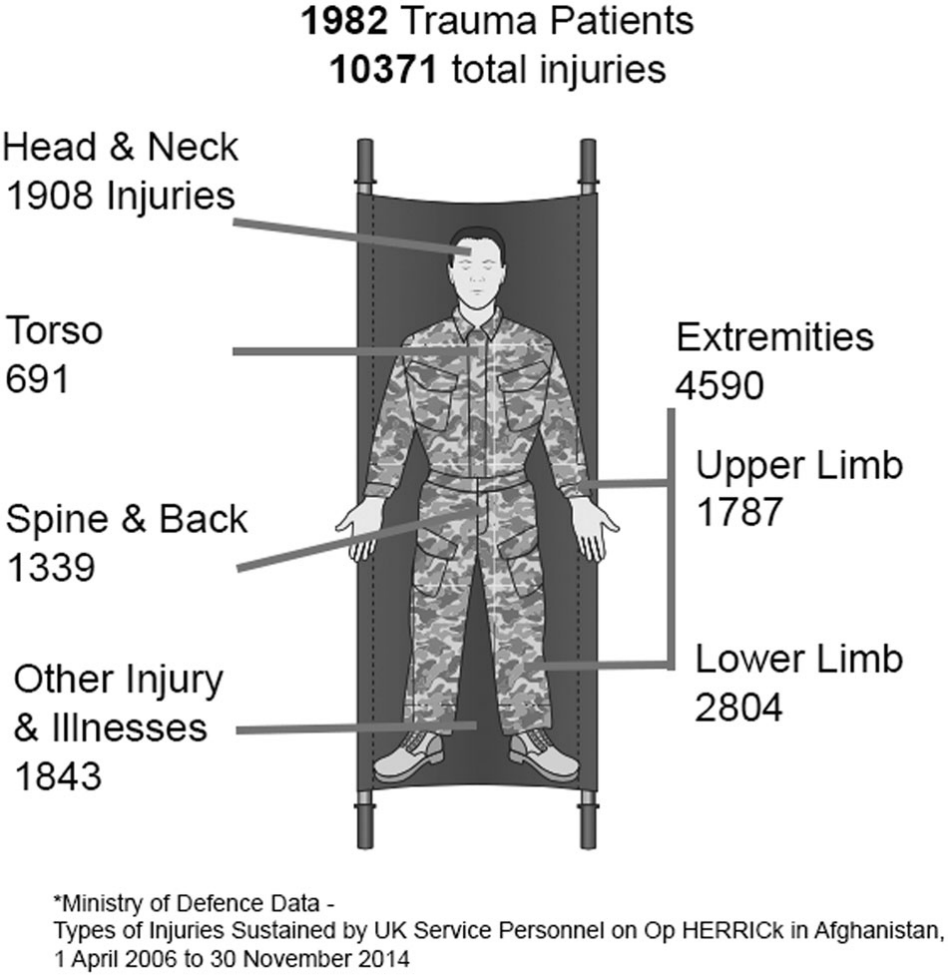
Diagram detailing injury types and prevalence from the most recent conflict in Afghanistan. Data from ‘Types of Injuries Sustained by UK Service Personnel on Op HERRICK in Afghanistan’ produced by the UK Ministry of Defence: https://assets.publishing.service.gov.uk/government/uploads/system/uploads/attachment_data/file/502888/20160223_Afghanistan_Types_of_Injuries_Official_Statistic_Final_OS.pdf

**Fig. 2:**
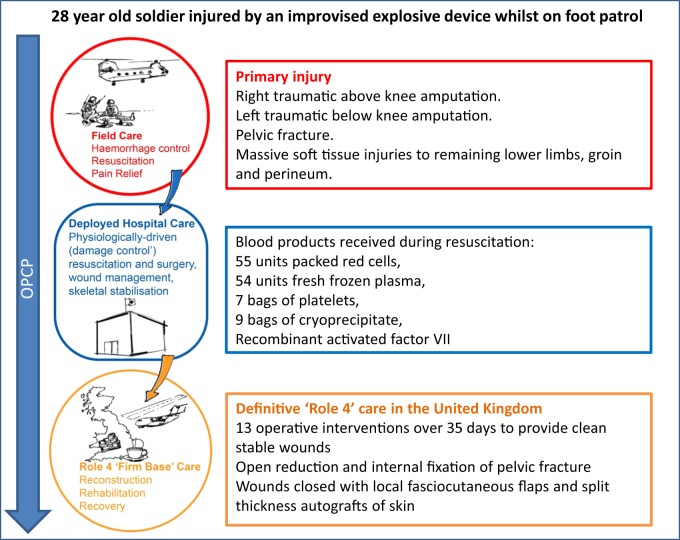
Diagram detailing an illustrative clinical case from the most recent conflict in Afghanistan with associated representation of the Operational Patient Care Pathway (OPCP) for that patient. Diagram drawn and assembled by John Skinner (Dstl Imagery) and authors. Further details on the nature of complex, multifaceted blast injury can be found in the comprehensive review by Cannon et al. including representative photographs of injuries^13^

Severe trauma has a diverse aetiology. There are, however, certain features common to most cases, including haemorrhage, the presence of hard and soft tissue damage, and pain. Trauma sustained during combat has some additional and unique facets. These can be divided into: (a) the mechanism and severity of injury (which do share some commonalities with civilian terrorist or ‘active shooter’ incidents), and; (b) the environment and the logistical context in which injuries are managed. It is the combination of these two facets that creates a uniquely challenging demand on deployed combat trauma care as described below. Figures 1 and 2 are included to further illustrate this. Figure 1 details injury types and prevalence from the recent conflict in Afghanistan while Fig. 2 describes a specific clinical case from that conflict.

### Mechanism and severity of injury

The mechanism of injury produced by military munitions often generates injuries of a greater number and of a far higher severity than the relatively low energy mechanisms of, for example, a fall from height or a motor vehicle collision.^10^ Injuries often involve the loss of a large volume of tissue which may include traumatic amputation of more than one limb.^11^ While a multitude of wounding modalities occur on a battlefield, penetrating injuries from high velocity small arms and blast injuries resulting from explosive ordnance predominate. Blast injury is increasingly prevalent, causing approximately 80% of modern combat injury.^12^ Further details on the nature of complex, multifaceted blast injury can be found in the comprehensive review by Cannon et al.^13^

Combat wounds are often highly contaminated that further contributes to the systemic inflammatory response associated with severe tissue damage. Wounds are often subject to further tissue loss as a result of repeated surgical attempts to remove infected and progressively devitalised tissues and the dysregulation of molecular pathways for cell proliferation, survival, and wound remodelling.

### Medical management

Some key differences from civilian trauma care exist in the context in which wartime trauma care is delivered. These can be divided into the time taken to reach medical care, the burden of ongoing severely injured casualties, the complexities of casualty treatment across internationally dispersed and internationally delivered military trauma systems, and the constraints created by limited resources.

#### Time to professional care

For a casualty suffering major trauma in London, the average time taken to arrive at a Major Trauma Centre is 17 min after injury. Virtually all patients reach one within 45 min.^14^ In contrast, prehospital timelines on the battlefield can be very protracted. World War II saw long evacuation times, of an average in the region of 12.5 h.^15^ During the Korean War casualty evacuation times were around 5 h or more, depending on which injuries were analysed.^16^ The Falklands War of 1982 saw highly variable casualty evacuation times, often of over 8 h.^17^ In contrast, time to professional medical care in Helmand Province, during the period July 2008–March 2012 averaged 75 min.^18^ Time to delivery of care is important. In recent conflicts, the delivery of damage control surgery within an hour of injury has been shown to save lives.^19^

#### Burden of ongoing casualties

With some notable exceptions, the majority of peacetime civilian trauma occurs as a single, isolated incident, and results in a relatively small number of casualties with severe injuries. In contrast, military doctors are often required to deal concurrently and repeatedly with a greater number of severely injured. Triage of the injured is frequently required and decisions about salvagability must be made that balance the compounding factors of limited time, equipment, supplies, personnel and evacuation capabilities—adopting a ‘do the most for the most’ approach.

#### Trauma system dispersal

Trauma care is most effective when delivered as part of an organised system.^20^ The commissioning of major trauma centres and networks in England has resulted in a measureable and significant improvement in survival.^21^ Similarly, deployed trauma systems have been shown to be effective, despite the fact that war is both chaotic and dynamic.^22^ British casualties of conflict move through a trauma system designed to provide stepwise increments in capability and capacity, from immediate buddy−buddy care, through enhanced first aid delivered by a ‘medic’ embedded within a combat unit or on board a warship, and the en route delivery of care on medical retrieval platforms to more fixed medical treatment facilities (MTFs) in echelons of increasing capability and capacity, ultimately ending in National Health Service hospitals in the United Kingdom. The potential geographical dispersal of this ‘Operational Patient Care Pathway’ is well illustrated by the Falklands War, where casualties were moved by land or by helicopter from point of wounding to forward surgical facilities, before being flown offshore to the hospital ship SS *Uganda*, a flight time of up to 40 min.^17^ Following further treatment on board SS *Uganda*, British injured were transported by other ships to Uruguay, a journey of 1000 nautical miles taking 6 days, before being flown back to the UK in specially equipped aeromedical evacuation platforms.^23^ Providing care en route allows our injured to be treated without pause, exemplified in the conflict in Afghanistan by the Royal Air Force’s CCAST capability.^24^

#### Logistics

Military medical logistics are multidimensional, requiring anticipation of the distributive nature or forward advance of battle, the safety of evacuation routes, as well as a proximity to the sea or air ports from which casualties will be evacuated home. Land-based MTFs deploy packed in ISO containers that, in the main, require transport by sea and subsequent assembly over 7−14 days. Ship-based MTFs in contrast remain intact ready to become operational within hours of arrival in theatre. Equipment must be robust enough to survive transportation and use within either environment. Resupply chains for such facilities cannot be assumed to be secure, or reliable. Medical materiel should ideally be light, physically robust and able to withstand prolonged storage at an extreme range of temperatures.

The unique challenges involved in delivering high-quality deployed trauma care, combined with the moral imperative to care for those injured in combat propagates a higher appetite for risk. This engenders early adoption of innovative approaches and lessons learnt from research. At the end of conflict, developments in military medicine are invariably taken up within civilian trauma care, taking them out of their military niche and broadening their utility.^25^

## Focusing defence requirements in regenerative medicine

**Fig. 3:**
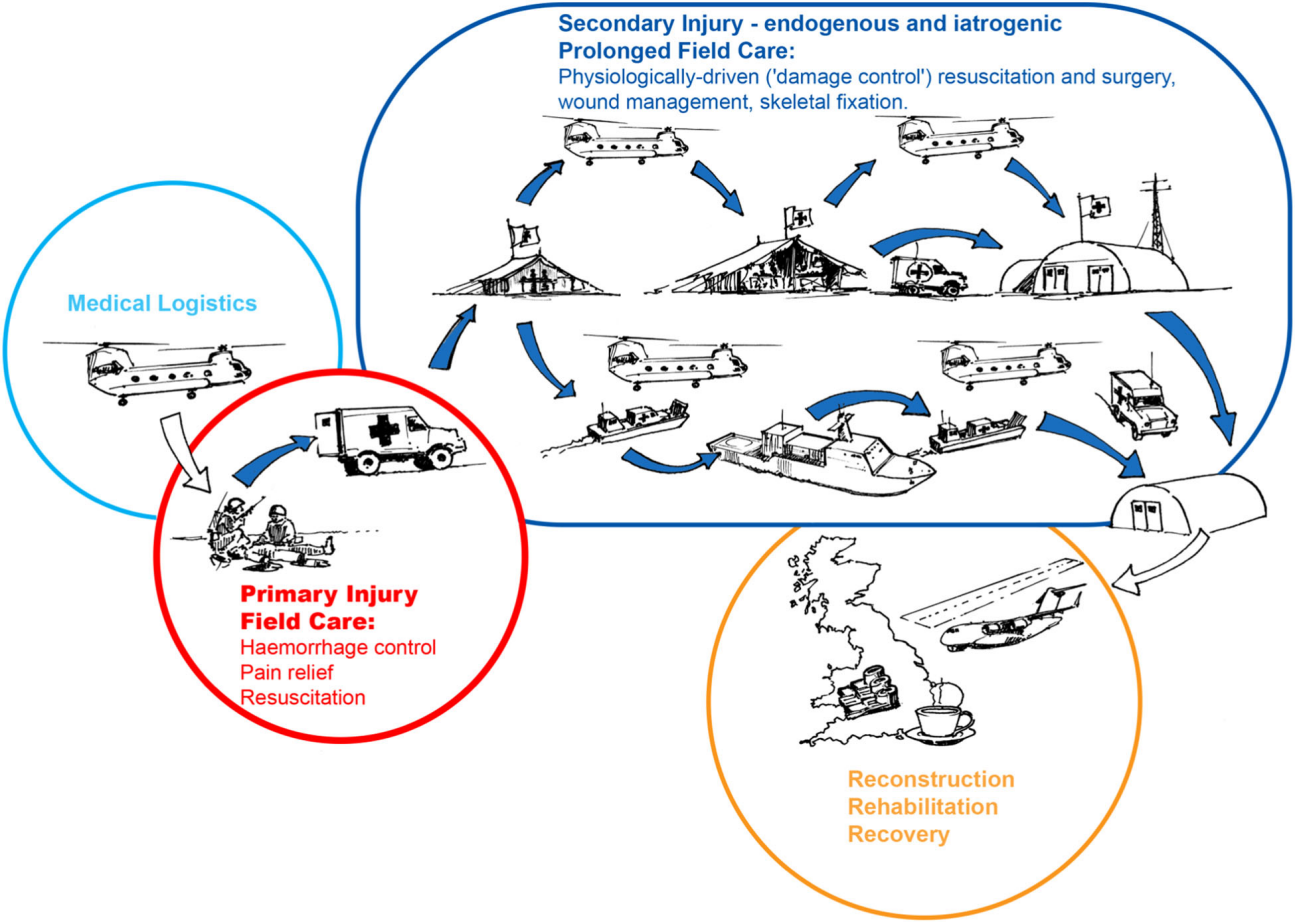
Depiction of possible future scenarios for the Operational Patient Care Pathway (OPCP), derived from the Allied Joint Doctrine on Medical Management and future operational analysis. The OPCP has been split into four broad sections: the supply of medical capability (logistics), far-forward care at the point of primary injury, a period of potential secondary injury within Deployed Medical Treatment Facilities, either iatrogenic in nature (e.g. wound debridement) or a feature of the endogenous response to trauma (e.g. apoptotic cell death), and finally, reconstruction, recovery and rehabilitation in the Firm Base. Diagram drawn and assembled by John Skinner (Dstl Imagery) and authors

The management of combat-related trauma presents some unique requirements for the research community, but how, specifically, might approaches in the field of regenerative medicine be applied to the aforementioned challenges? In focusing our needs, the authors sought opinion from clinical and academic leaders across specialities within the British Defence Medical Services, through online surveys and workshops. The conclusions from this engagement were analysed in the context of the current regenerative medicine research landscape in the UK and globally discerned via literature searches, meetings and conference attendance. The combined approach to this scoping study followed the methodology of Arksey and O’Mally.^26^ Details can be found in Supplementary Methods. Our conclusions from this study are:

- while a significant amount of high-quality regenerative medicine research is ongoing in UK and elsewhere, significant research gaps exist in areas that would meet UK operational research requirements, and;
- regenerative approaches are applicable across the Operational Patient Care Pathway (OPCP, Fig. 3).^25^

As discussed in the introduction, future conflicts may involve more complicated and protracted medical evacuation than those experienced recently in Afghanistan. A greater burden of patient management may therefore fall in the prehospital phase, with a requirement to perform life-saving interventions and prevent physiological deterioration, while simultaneously evacuating a casualty over highly extended timelines. This may need to be done with minimal logistic support under austere conditions (e.g. extreme weather conditions, limited access to electrical power).

In this context it has become clear, through the process of our scoping study, that while the use of regenerative medicine strategies at the later stages of the OPCP constitutes the status quo, and a focus on reconstructive tissue engineering can be influenced outside of Defence, there is an exciting opportunity to investigate their far-forward application.

In the subsequent sections we lay out three research themes where regenerative medicine approaches could be applied at these very early time points and have the potential to provide a step-change in care, particularly for complex blast injury, with benefits for later functional recovery. Although it has been necessary to discuss example technologies for the purpose of explanation we have sought to keep discussions as nonspecific as possible to allow flexibility of thought around potential regenerative solutions.

## Front-line focus

### Bioengineered blood

The use of blood products has been an important theme in the management of severely wounded personnel. The First World War was the catalyst for the development of blood banks and improved transfusion techniques,^27^ and the relationship between war and innovation in transfusion medicine continues today. Haemorrhage is still the leading cause of ‘potentially salvageable’ death on the battlefield.^28^ Evidence from conflicts, both recent and distant, suggests that the use of blood products before reaching hospital, in both civilian and military scenarios, provides benefit.^29–31^ Despite this, challenges remain in the appropriate storage and delivery of these high-value interventions in austere environments.^32^ The tools and techniques of regenerative medicine will likely hold a key to the logistical challenges of using blood products in future conflicts (for example, the production of blood cells from stem cell populations and the manipulation of components produced in vitro for reduced immunogenicity and improved storage profiles). Over and above this, however, blood components could be used as regenerative tools themselves and combining these concepts could provide truly disruptive solutions for Defence.

#### Red blood cells

Red blood cells (RBCs) must be acquired currently from donor individuals, and undergo appropriate safety testing and cross-matching procedures. To address these safety concerns, interest has grown in the use of wholly synthetic components of blood, particularly for oxygen carriage. As yet, however, these have not been adopted into clinical use. The potential for blood components to be manufactured in vitro at scale is growing. Indeed, the first infusion of cultured RBCs in a human was described in 2011.^33^ RBCs are being produced from a variety of sources,^34–36^ although significant challenges remain in scalability, reproducibility and cost.^37^ Recently, a human erythroid cell line has been developed which may lay further foundation for the generation of universally ABO-compatible RBCs in vitro.^38^

#### Platelets

Platelets are given as a component of blood transfusion protocols under current British military transfusion guidelines.^39,40^ Warm-stored platelets have a short shelf-life (5 days) and so in a military situation they must be frequently flown to forward MTFs.^41^ As for RBCs, large-scale manufacture of platelets in vitro from stem cells would obviate the need for blood donors and ensure a ready supply. Recent efforts to produce platelets in vitro have achieved some success, although still with low yield and functional limitations. In addition, challenges in producing consistent, defined populations remain.^42^ Tissue engineering techniques applied to platelets to produce a product with a longer shelf would have particular utility.

For much of the time since their discovery, platelets have been viewed solely as mediators of coagulation. More recently, however, it has been shown that platelets can have important functions within the immune system and in tissue regeneration.^43,44^ It is becoming apparent that different populations of platelets exist depending on spatial, temporal and physical factors and indeed, their multifunctional nature has been likened to a Swiss army knife.^45^ Production of platelets in vitro may allow these different functions to be harnessed and tailored. For example, platelet-derived SDF-1 was shown to potentiate lung regeneration after pneumonectomy.^43^ SDF-1 signalling is key for the mobilisation of cells from the bone marrow and modulation of the SDF-1/CXCR4 axis has been investigated as a way of enhancing the endogenous regenerative response after injury.^46^ Different populations of platelets could be engineered and selected for different functions after trauma.

Tissue engineering requires a scaffold and in the case of blood that ‘scaffold’ is plasma. Plasma itself can be given alone as a prehospital intervention after severe haemorrhage^47^ or, more commonly, as part of component therapy.^48^ As for RBCs and platelets its use in the prehospital arena brings logistical challenges. A number of nations, including France, Israel and Germany, use dried plasma products in military or austere civilian situations.^49^ The Israeli Defense Force have implemented, and demonstrated efficacy of, the use of freeze-dried plasma from the point of wounding.^47^ While plasma is currently obtained from donors, future advances may also obviate the need for donation if a cost-effective synthetic substitute can be developed or donor plasma be engineered for additional functionality.

Packed red cells and platelets have been the only ‘cellular therapy’ used on combat operations in recent conflicts, as replacements for blood lost as a result of haemorrhage. Other cell therapies may also have utility far forward if logistical hurdles are surmounted.^50^ Advances in the production of these components in vitro now also opens up opportunities in ‘tissue’ engineering of blood products to engender additional or tailored functionality (including proregenerative) and stability, thus providing therapeutic and logistical advantages.

### The exploitation of mechanobiology and other physical phenomena for wound regeneration

The repair and regeneration of severe tissue damage involves a complex set of biological processes involving different structures including vasculature, muscle and neural tissue. A fine balance of appropriate biological responses must occur for successful functional regeneration and reconstruction. Impaired wound healing and regeneration are generally associated with the dysregulation of these biological processes. The mediators involved, including growth factors, cytokines and progenitor cells, have therefore become the focus of wound therapeutics. Biological strategies to aid wound repair and regeneration have largely dominated the wound research area with some successes, including in severe muscle injury.^51–53^ These approaches, however, present logistical difficulties in austere environments and may be hampered by ongoing local and systemic processes.

Over recent years it has become apparent that the regeneration of composite tissue defects (whether endogenous or exogenously supported) depends not only on biological properties, but also on the physical environment.^54^ Our understanding, however, of how the biophysical properties of a wound affect progressive tissue loss and subsequent regeneration is still relatively nascent, particularly with respect to the level of volumetric tissue loss often associated with traumatic wounds. Combining an understanding of the mechanobiology of relevant wounds with materials science, including nanotechnologies, bioengineering and biophysics could enable the development of novel, logistically light, early wound management strategies that maintain cellular viability and enhance the regenerative potential of cells within the wound bed during casualty evacuation.

Research directed at this area has increased in recent years. Novel biomaterials for wound management are being optimised for surface chemistry, topography and therapeutic loading, and made from a variety of synthetic and natural substances in different physical manifestations (e.g. gels, sprays, powders and scaffolds).^55–57^ The principle focus of the development of novel regenerative wound materials has been on reconstruction and definitive wound management, but it may well hold true that some of these principles and materials can be applied much earlier.^58^

The application of mechanical forces on wound tissue may also provide a mechanism to modulate wound regeneration.^58^ The physical changes brought about by current treatment regimens (e.g. topical negative pressure) and how these might affect regeneration are not fully understood.^59^ An enhanced understanding of the mechanobiology of relevant wounds, particularly in injured muscle, may lead to optimised versions of these already-fielded approaches.

Beyond novel materials or mechanical manipulation, there is also evidence that wound repair and regeneration can be influenced by other physical phenomena such as light,^60^ acoustic waves,^61^ or electrical stimulation,^62^ which may also have utility in early wound management.

Where research in these areas has transitioned in vivo, it has largely focussed on the healing of recalcitrant chronic wounds or, in the case of acute wounding, small and minimally disruptive wounds. It is difficult to extrapolate these findings to severe traumatic wounds, whatever the therapeutic modality. In addition, it is important that outcomes focus on wound regeneration, rather than simply on wound repair.^63^

Definitive wound management and reconstruction are unlikely to take place in the field, and the approaches detailed here cannot engender full regeneration in the time taken for evacuation, however protracted. Various wound management techniques are, however, used at these acute time points, including the use of topical negative pressure.^64,65^ A better understanding of the areas highlighted above may allow optimisation of such current treatments and precipitate the development of novel engineered materials, methods and devices for modulating the physical properties of a wound, mitigating progressive cell death and stimulating regenerative potential.

### Harnessing the endogenous regenerative response in severe traumatic injury

In humans most tissues within the body have capacity for physiological regeneration, replacing cells lost as part of their natural life cycle. Most tissues, however, have a more limited capacity for reparative regeneration after injury. Furthermore, these regenerative functions decline with age.^66^ The ability to regenerate tissues after injury varies considerably between species in the animal kingdom, as does susceptibility of these responses to aging.^67^ There is likely to be a great deal we can learn about tissue regeneration from other species, and our own developmental biology.

Endogenous regeneration (reparative and physiological) can be executed, or mediated, by cells within the local environment (for example the regeneration of the liver through proliferation of existing differentiated hepatic cells),^68^ as well as cells that migrate from the bone marrow or peripheral circulation, for example the interplay between endothelial progenitor cells, fibroblasts and tissue-resident endothelial cells in vascular regeneration.^69^ Regenerative responses are also dependent on context. The limited regenerative capacity of many tissues, and their capacity for induced or supported regeneration, can be further complicated by the local and systemic environments. As an example, the metabolic deregulation that occurs in diabetes leads to dysfunctional stem and progenitor cell processes, which have been related to various diabetic complications, including a propensity to develop chronic, nonhealing wounds.^70^

The burden of tissue damage associated with severe traumatic injury can lead to a dysregulation of a variety of systems including immune, metabolic and haemostatic responses.^39,71,72^ Blast injury is also likely to produce particular direct molecular and cellular changes, exacerbated by deficient tissue oxygen delivery if blast-induced lung damage is present.^73^ A better understanding of this context and what happens to the viability, production, differentiation and migration of cells involved in repair and regeneration after severe trauma will be important for the development of novel early regenerative strategies, and may hold the key to understanding the phenomenon of progressive necrosis of wounded tissue. Links between the broader molecular and cellular context and the capacity for endogenous or supported regeneration are being made in a variety of conditions,^74^ but significant gaps in our understanding in severe trauma remain. Recent progress has been made in understanding systemic changes after injury, but a link to long-term regenerative potential has yet to be truly chronicled or understood and the leap to new therapeutics based on this understanding is still to follow.

Bone marrow dysfunction, in the form of prolonged mobilisation of hematopoietic progenitor cells, has been well-defined after trauma/haemorrhagic shock in both animal models and in patients,^75^ and has been linked to persistent anaemia and multiorgan failure. This effect has been successfully reversed by early β-receptor blockade, although this could not be linked to a statistically significant clinical outcome when trialled in patients.^76^ In animal models, a link was determined between this bone marrow dysfunction and reduced healing outcomes from a lung contusion, although this may not translate to other wounds.^77^ Outcomes related to wound healing or successful reconstruction were not present in the clinical study and it would be interesting to examine the effect of bone marrow dysfunction on the endogenous, or supported, regenerative capacity of different tissues.

Other cells within the bone marrow also have roles in tissue repair and regeneration, some of which have been studied after trauma, including the differentiation and mobilisation of granulocytes.^78–80^ There is, however, a great deal more to learn about the effect of severe trauma on the molecular and cellular components of regeneration and how this links to the later regenerative capacity of various tissues.

An appropriate balance of immune responses is also required for successful reparative tissue regeneration and it may be surmised that the huge immune perturbations after severe trauma might be detrimental to regeneration. Therapeutic modulation of such a complex system, however, is not straightforward. Successful tissue repair and regeneration often rely on appropriately timed inflammation. For example, M2 polarised macrophages have proven effective in reducing inflammation in a number of conditions, but early treatment of full-thickness excisional skin wounds in mice with M2 polarised macrophages did not provide a healing benefit, despite the cells exhibiting an anti-inflammatory profile in vitro.^81^ In this case it was postulated that early proinflammatory signals were necessary for appropriate wound healing and that it was a prolonged proinflammatory response that was detrimental. This demonstrates that while the modulation of the inflammatory response after trauma shows great potential as a mechanism to potentiate tissue regeneration, it requires balance and timing. Therapeutics that are able to respond to the inflammatory milieu may also prove important.

As well as providing insight into how best to support endogenous and induced regeneration following trauma, a better understanding of the molecular and cellular changes that affect tissue regeneration may also shed further light on the phenomenon of progressive tissue deterioration after traumatic insult. This observed phenomenon is being studied after traumatic brain injury in a search for neuroprotective and regenerative therapies.^82^ Significantly less is known in the context of severe musculoskeletal injury. For many chronic wounds, a permanent inflammatory state results in the degradation of newly forming tissue, which creates a vicious cycle.^83^ Tissue deterioration has been linked to an inappropriate balance of proteases and their inhibitors in both acute and chronic wounds, including combat wounds.^83,84^ Elevated levels of these enzymes are often measured days, weeks or months after initial development of the wound; however, a molecular assessment of the wound in its early stages might elucidate the initiating mediators of the spiral of deterioration.

It is clear that successful regeneration of any tissue is dependent on context. It has been noted that regenerative medicine must not only be about providing injured tissues with the therapy, whether that be stem and progenitor cells, or a novel regenerative material, but also with an environment conducive to regeneration.^67^ In the case of combat trauma there is more to learn about the regenerative context of tissues, and the effect of protracted evacuation. Early manipulation of this environment is likely to provide benefit to later regenerative and reconstructive processes.

### Models for testing early regenerative therapies for traumatic injury

Having defined the research priorities detailed in this paper we have also embarked on a series of activities to support research in these areas. In doing so, however, we have recognised that an important, underpinning requirement is the availability of appropriate models in which future therapies can be tested. In the case of testing engineered blood components for their resuscitative functions, a variety of militarily-relevant models of poly-trauma exist using terminally anesthetised animals, including in our own lab.^85,86^ Ultimately however, in order to appraise the direct regenerative effect of a therapy, or its influence on later regenerative and reconstructive practices, longer term recovery models will be required. Models of relevant extremity soft tissue injury are rare or have been developed for testing alternative therapeutics (e.g. antimicrobial dressings).^87^ A number of models of ‘volumetric tissue loss’ exist, but the lesion is usually generated surgically and there is no additional element to model the associated physiological or immunological response to severe trauma.^88–90^ Other models of extremity skeletal muscle trauma do look to replicate the physiological response to trauma (for example, muscle necrosis and ischaemia) without necessarily recreating exact injury mechanisms.^91,92^ Thus far these have been used to evaluate tissue engineering therapies more suited to a definitive care setting. There are many other examples of models of less severe acute wounding in a variety of species but many of these models are not optimal and do not adequately represent the nature of military traumatic injury. Much as described for the development of novel therapeutics for chronic wounds by Ansell et al., the development of novel therapeutic avenues for potentiating regeneration in severe traumatic wounds is a ‘catch 22’ situation: the identification of novel therapies is impossible without a range of suitable validated models but the development of such models is somewhat hampered without a purpose for developing them and positive controls for validation.^93^ The testing of novel therapeutics should, of course, progress through a series of increasingly complex models; however, there are currently steps in the sequence missing or requiring modification. The full range of tools should be appraised including the use of in vitro and ex vivo models such as perfused systems and organoids.^94^

## Conclusion

Severe combat traumatic injury involves the loss of, and damage to, tissues and organ systems through direct and indirect mechanisms. More than one tissue type or organ system is usually involved. Approaches to regenerate or replace these structures, with a particular focus on functional regeneration must therefore be a central tenet of successful recovery. Severe injury sustained during conflict has unique facets that provide additional contextual challenges to the application of regenerative strategies but also, we suggest, some unique, disruptive, opportunities. Through a detailed scoping study, we have concluded that regenerative medicine-inspired solutions could be applied across the Operational Patient Care Pathway. We have identified specific research themes that would engender the development of regenerative solutions for improved functional recovery from injuries sustained in conflict, namely bioengineered blood, the mechanobiology of blast and ballistic wounding, and modifying the effect of severe traumatic injury on endogenous regenerative potential.

In seeking logistically light ways to not only halt the progression of secondary injury but to generate an early proregenerative agenda, we believe there is the potential for improved functional recovery for our injured.

© Crown copyright (2017), Dstl and Royal Centre for Defence Medicine. This material is licensed under the terms of the Open Government Licence except where otherwise stated. To view this licence, visit http://www.nationalarchives.gov.uk/doc/open-governmentlicence/version/3 or write to the Information Policy Team, The National Archives, Kew, London TW9 4DU, or email: psi@nationalarchives.gsi.gov.uk.

## Electronic supplementary material

Supplementary methods for scoping study

## Electronic supplementary material

**Supplementary information** accompanies the paper on the *npj Regenerative Medicine* website (10.1038/s41536-018-0053-4).

## References

[1] Penn-Barwell JG, Roberts SA, Midwinter MJ, Bishop JR (2015). Improved survival in UK combat casualties from Iraq and Afghanistan: 2003−2012. J. Trauma Acute Care Surg..

[2] Ministry of Defence. Op Herrick casualty and fatality tables to 28 February 2015. https://www.gov.uk/government/statistics/op-herrick-casualty-and-fatality-tables-2015 (2016).

[3] Keene DD (2016). Died of wounds: a mortality review. J. R. Army Med. Corps.

[4] DeBruyne, N. F. *American War and Military Operations Casualties: Lists and Statistics* (Congressional Research Service, Washington, DC, 2017).

[5] Evriviades D (2011). Shaping the military wound: issues surrounding the reconstruction of injured servicemen at the Royal Centre for Defence Medicine. Philos. Trans. R. Soc. Lond. B Biol. Sci..

[6] Murray CJ (2012). Disability-adjusted life years (DALYs) for 291 diseases and injuries in 21 regions, 1990−2010: a systematic analysis for the Global Burden of Disease Study 2010. Lancet.

[7] Haagsma JA (2016). The global burden of injury: incidence, mortality, disability-adjusted life years and time trends from the Global Burden of Disease study 2013. Inj. Prev..

[8] Krug EG, Sharma GK, Lozano R (2000). The global burden of injuries. Am. J. Public Health.

[9] Findlay, G. et al. *Trauma: Who Cares?* (National Confidential Enquiry into Patient Outcome and Death, 2007).

[10] Champion HR, Bellamy RF, Roberts CP, Leppaniemi A (2003). A profile of combat injury. J. Trauma.

[11] Chandler H, MacLeod K, Penn-Barwell JG, Severe Lower Extremity Combat Trauma Study Group. (2017). Extremity injuries sustained by the UK military in the Iraq and Afghanistan conflicts: 2003−2014. Injury.

[12] Owens BD (2008). Combat wounds in operation Iraqi Freedom and operation Enduring Freedom. J. Trauma.

[13] Cannon JW (2016). Dismounted complex blast injuries: a comprehensive review of the modern combat experience. J. Am. Coll. Surg..

[14] McCullough AL, Haycock JC, Forward DP, Moran CG (2014). Major trauma networks in England. Br. J. Anaesth..

[15] Debakey ME, Simeone FA (1946). Battle injuries of the arteries in World War II: an analysis of 2,471 cases. Ann. Surg..

[16] Hughes CW (1958). Arterial repair during the Korean war. Ann. Surg..

[17] Jackson DS, Batty CG, Ryan JM, McGregor WS (2007). Army field surgical experience. J. R. Army Med. Corps.

[18] Morrison JJ (2013). En-route care capability from point of injury impacts mortality after severe wartime injury. Ann. Surg..

[19] Kotwal RS (2016). The effect of a golden hour policy on the morbidity and mortality of combat casualties. JAMA Surg..

[20] Mullins RJ (1999). A historical perspective of trauma system development in the United States. J. Trauma.

[21] NHS England. Independent review of Major Trauma Networks reveals increase in patient survival rates. https://www.england.nhs.uk/2013/06/incr-pati-survi-rts/ (2013).

[22] Eastridge BJ (2009). Impact of joint theater trauma system initiatives on battlefield injury outcomes. Am. J. Surg..

[23] Penn-Barwell JG, Jolly RT, Rickard RF (2017). Medical support to operation Corporate. J. R. Nav. Med Serv..

[24] Turner S, Ruth M, Tipping R (2009). Critical care air support teams and deployed intensive care. J. R. Army Med. Corps.

[25] Gulland A (2008). Emergency medicine: lessons from the battlefield. BMJ.

[26] Arksey H, O’Malley L (2005). Scoping studies: towards a methodological framework. Int. J. Social Res. Methodol..

[27] Boulton F, Roberts DJ (2014). Blood transfusion at the time of the First World War—practice and promise at the birth of transfusion medicine. Transfus. Med..

[28] Ravi PR, Puri B (2017). Fluid resuscitation in haemorrhagic shock in combat casualties. Disaster Mil. Med.

[29] Lyon RM (2017). Pre-hospital transfusion of packed red blood cells in 147 patients from a UK helicopter emergency medical service. Scand. J. Trauma, Resusc. Emerg. Med..

[30] Watts S (2015). Evaluation of prehospital blood products to attenuate acute coagulopathy of trauma in a model of severe injury and shock in anesthetized pigs. Shock.

[31] Beecher HK (1945). Preparation of battle casualties for surgery. Ann. Surg..

[32] Neuhaus SJ, Wishaw K, Lelkens C (2010). Australian experience with frozen blood products on military operations. Med. J. Aust..

[33] Giarratana MC (2011). Proof of principle for transfusion of in vitro-generated red blood cells. Blood.

[34] Olivier EN, Qiu C, Velho M, Hirsch RE, Bouhassira EE (2006). Large-scale production of embryonic red blood cells from human embryonic stem cells. Exp. Hematol..

[35] Douay L, Andreu G (2007). Ex vivo production of human red blood cells from hematopoietic stem cells: what is the future in transfusion?. Transfus. Med. Rev..

[36] Dias J (2011). Generation of red blood cells from human induced pluripotent stem cells. Stem Cells Dev..

[37] Giarratana MC, Marie T, Darghouth D, Douay L (2013). Biological validation of bio-engineered red blood cell productions. Blood Cells Mol. Dis..

[38] Trakarnsanga K (2017). An immortalized adult human erythroid line facilitates sustainable and scalable generation of functional red cells. Nat. Commun..

[39] Mercer SJ, Tarmey NT, Woolley T, Wood P, Mahoney PF (2013). Haemorrhage and coagulopathy in the Defence Medical Services. Anaesthesia.

[40] Doughty HA, Woolley T, Thomas GO (2011). Massive transfusion. J. R. Army Med. Corps.

[41] Hess JR, Lelkens CC, Holcomb JB, Scalea TM (2013). Advances in military, field, and austere transfusion medicine in the last decade. Transfus. Apher. Sci..

[42] Wang Y (2015). Comparative analysis of human ex vivo-generated platelets vs megakaryocyte-generated platelets in mice: a cautionary tale. Blood.

[43] Rafii S (2015). Platelet-derived SDF-1 primes the pulmonary capillary vascular niche to drive lung alveolar regeneration. Nat. Cell Biol..

[44] Nurden AT (2011). Platelets, inflammation and tissue regeneration. Thromb. Haemost..

[45] Reviakine I (2015). New horizons in platelet research: understanding and harnessing platelet functional diversity. Clin. Hemorheol. Microcirc..

[46] Rennert RC, Sorkin M, Garg RK, Gurtner GC (2012). Stem cell recruitment after injury: lessons for regenerative medicine. Regen. Med..

[47] Shlaifer A (2017). Prehospital administration of freeze-dried plasma, is it the solution for trauma casualties?. J. Trauma Acute Care Surg..

[48] Holcomb JB (2015). Transfusion of plasma, platelets, and red blood cells in a 1:1:1 vs a 1:1:2 ratio and mortality in patients with severe trauma: the PROPPR randomized clinical trial. Jama.

[49] Pusateri AE (2016). Dried plasma: state of the science and recent developments. Transfusion.

[50] Pati S (2015). Cellular therapies in trauma and critical care medicine: forging new frontiers. Shock.

[51] Barrientos S, Brem H, Stojadinovic O, Tomic-Canic M (2014). Clinical application of growth factors and cytokines in wound healing. Wound Repair Regen..

[52] Passipieri JA, Christ GJ (2015). The potential of combination therapeutics for more complete repair of volumetric muscle loss injuries: the role of exogenous growth factors and/or progenitor cells in implantable skeletal muscle tissue engineering technologies. Cells Tissues Organs.

[53] Wu Y, Wang J, Scott PG, Tredget EE (2007). Bone marrow-derived stem cells in wound healing: a review. Wound Repair Regen..

[54] Rosinczuk J, Taradaj J, Dymarek R, Sopel M (2016). Mechanoregulation of wound healing and skin homeostasis. Biomed. Res. Int..

[55] Sandri G (2017). Halloysite and chitosan oligosaccharide nanocomposite for wound healing. Acta Biomater..

[56] Ghadiri M, Chrzanowski W, Rohanizadeh R (2014). Antibiotic eluting clay mineral (Laponite(R)) for wound healing application: an in vitro study. J. Mater. Sci. Mater. Med..

[57] Sridharan R, Cameron AR, Kelly DJ, Kearney CJ, O’Brien FJ (2015). Biomaterial based modulation of macrophage polarization: a review and suggested design principles. Mater. Today.

[58] Thompson WR, Scott A, Loghmani MT, Ward SR, Warden SJ (2015). Understanding mechanobiology: physical therapists as a force in mechanotherapy and musculoskeletal regenerative rehabilitation. Phys. Ther..

[59] Huang C, Leavitt T, Bayer LR, Orgill DP (2014). Effect of negative pressure wound therapy on wound healing. Curr. Probl. Surg..

[60] Kuffler DP (2016). Photobiomodulation in promoting wound healing: a review. Regen. Med..

[61] Mittermayr R (2012). Extracorporeal shock wave therapy (ESWT) for wound healing: technology, mechanisms, and clinical efficacy. Wound Repair Regen..

[62] Ashrafi M, Alonso-Rasgado T, Baguneid M, Bayat A (2017). The efficacy of electrical stimulation in lower extremity cutaneous wound healing: a systematic review. Exp. Dermatol..

[63] You HJ, Han SK (2014). Cell therapy for wound healing. J. Korean Med. Sci..

[64] Penn-Barwell JG, Fries CA, Street L, Jeffery S (2011). Use of topical negative pressure in British servicemen with combat wounds. Eplasty.

[65] Leininger BE, Rasmussen TE, Smith DL, Jenkins DH, Coppola C (2006). Experience with wound VAC and delayed primary closure of contaminated soft tissue injuries in Iraq. J. Trauma.

[66] Yun MH (2015). Changes in regenerative capacity through lifespan. Int. J. Mol. Sci..

[67] Coffman JA, Rieger S, Rogers AN, Updike DL, Yin VP (2016). Comparative biology of tissue repair, regeneration and aging. npj Regen. Med..

[68] Kholodenko IV, Yarygin KN (2017). Cellular mechanisms of liver regeneration and cell-based therapies of liver diseases. Biomed. Res. Int..

[69] Zhang M, Malik AB, Rehman J (2014). Endothelial progenitor cells and vascular repair. Curr. Opin. Hematol..

[70] Rodrigues M (2015). Progenitor cell dysfunctions underlie some diabetic complications. Am. J. Pathol..

[71] Stoecklein VM, Osuka A, Lederer JA (2012). Trauma equals danger—damage control by the immune system. J. Leukoc. Biol..

[72] Lord JM (2014). The systemic immune response to trauma: an overview of pathophysiology and treatment. Lancet.

[73] Ganie FA (2013). Lung contusion: a clinico-pathological entity with unpredictable clinical course. Bull. Emerg. Trauma.

[74] Godwin JW, Pinto AR, Rosenthal NA (2017). Chasing the recipe for a pro-regenerative immune system. Semin Cell Dev. Biol..

[75] Livingston DH (2003). Bone marrow failure following severe injury in humans. Ann. Surg..

[76] Bible LE (2014). Early propranolol administration to severely injured patients can improve bone marrow dysfunction. J. Trauma Acute Care Surg..

[77] Hannoush EJ (2013). Role of bone marrow and mesenchymal stem cells in healing after traumatic injury. Surgery.

[78] Santangelo S, Gamelli RL, Shankar R (2001). Myeloid commitment shifts toward monocytopoiesis after thermal injury and sepsis. Ann. Surg..

[79] Moore FA, Peterson VM, Moore EE, Rundus C, Poggetti R (1990). Inadequate granulopoiesis after major torso trauma: a hematopoietic regulatory paradox. Surgery.

[80] Hampson P (2017). Neutrophil dysfunction, immature granulocytes, and cell-free DNA are early biomarkers of sepsis in burn-injured patients: a prospective observational cohort study. Ann. Surg..

[81] Jetten N (2014). Wound administration of M2-polarized macrophages does not improve murine cutaneous healing responses. PLoS ONE.

[82] Kumar A, Loane DJ (2012). Neuroinflammation after traumatic brain injury: opportunities for therapeutic intervention. Brain Behav. Immun..

[83] Trengove NJ (1999). Analysis of the acute and chronic wound environments: the role of proteases and their inhibitors. Wound Repair Regen..

[84] Utz ER (2010). Metalloproteinase expression is associated with traumatic wound failure. J. Surg. Res..

[85] Kirkman E, Watts S, Cooper G (2011). Blast injury research models. Philos. Trans. R. Soc. B: Biol. Sci..

[86] Watts S (2015). Evaluation of prehospital blood products to attenuate acute coagulopathy of trauma in a model of severe injury and shock in anesthetized pigs. Shock (Augusta, Ga.).

[87] Eardley WG (2012). The development of an experimental model of contaminated muscle injury in rabbits. Int. J. Low Extrem. Wounds.

[88] Li MTA, Willett NJ, Uhrig BA, Guldberg RE, Warren GL (2014). Functional analysis of limb recovery following autograft treatment of volumetric muscle loss in the quadriceps femoris. J. Biomech..

[89] Corona BT, Ward CL, Baker HB, Walters TJ, Christ GJ (2014). Implantation of in vitro tissue engineered muscle repair constructs and bladder acellular matrices partially restore in vivo skeletal muscle function in a rat model of volumetric muscle loss injury. Tissue Eng. Part A.

[90] Ward CL (2016). Autologous minced muscle grafts improve muscle strength in a porcine model of volumetric muscle loss injury. J. Orthop. Trauma.

[91] Wang L (2014). Minimally invasive approach to the repair of injured skeletal muscle with a shape-memory scaffold. Mol. Ther..

[92] Pumberger M (2016). Synthetic niche to modulate regenerative potential of MSCs and enhance skeletal muscle regeneration. Biomaterials.

[93] Ansell DM, Holden KA, Hardman MJ (2012). Animal models of wound repair: are they cutting it?. Exp. Dermatol..

[94] Ud-Din S, Bayat A (2017). Non-animal models of wound healing in cutaneous repair: In silico, in vitro, ex vivo, and in vivo models of wounds and scars in human skin. Wound Repair Regen..

