# Supplementary material for: Regenerative medicine and war: a front-line focus for UK defence
Source: NPJ Regen Med. 2018 Aug 21;3:13. doi: 10.1038/s41536-018-0053-4 (PMC6104070; doi:10.1038/s41536-018-0053-4)
Supplement: Supplementary file 1 — Supplementary methods for scoping study [file 41536_2018_53_MOESM1_ESM.pdf]

Supplementary methods for scoping study

Literature Searches (carried out by the Knowledge Management and Exploitation Group at Dstl Porton Down):

In order to investigate the main thrust of regenerative medicine research in the UK over the last five years a literature review was conducted using the following search strings (2011-2016):

- *TS=(regenerative medicine OR regenerative therapies OR stem cell development OR tissue engineering) AND CU=(united kingdom OR UK OR england OR scotland OR wales);*
- *TS=(regenerative medicine OR regenerative therapies OR stem cell development OR tissue engineering OR neur* regen* OR restor* tissue) AND CU=(united kingdom OR UK OR england OR scotland OR wales)*

Resulting publications were then ranked by UK institution to reveal the top 10 institutions for publishing in the regenerative medicine area over the last 5 years. Authors from these publications were also ranked to reveal the top 50 authors (by numbers of publications) publishing in this area.

A subsequent literature review was conducted to help ascertain the level of trauma-related regenerative medicine research compared to other indications (e.g. cancer or neurodegenerative disorders). This review was conducted using the Web of Science (WOS) and MEDLINE databases. The search terms “regenerative medicine,” “stem cell therapy,” and “tissue engineering” were combined with a range of terms to cover trauma (e.g. trauma*, wound*, burn*) and alternative indications (e.g. neuro*, cancer, diabetes). These searches were not limited to the UK.

Bibliometric analysis was conducted on a combined dataset which comprised of search results extracted from identical searches of WOS and MEDLINE. The references were merged, de-duplicated and cleaned to provide a single set of unique records. Analysis of references by country and author affiliation was undertaken.

These literature reviews found that trauma has not featured highly in the regenerative medicine literature of the last ten years. When the search term “trauma” is combined with

“regenerative medicine,” “tissue engineering,” or “stem cell therapy,” it forms around 2% of the overall literature for that search term in each case. This compares to 12% when “neuro” is combined with “regenerative medicine,” for example.

Already-collated information on the prevalence of regenerative medicine research by disease indication or organ system is scarce in the published literature itself. The level of trauma-focussed regenerative medicine research was also assessed using the other approaches detailed in these methods, including via other review and strategy documents. For example, the 2012 UK strategy on regenerative medicine assesses research council funding in regenerative medicine by disease indication and finds particular focus on generic research (e.g. safety, manufacturing) and musculoskeletal, and neurodegenerative disorders. In this review the percentage of regenerative medicine funding directed at “injury” (as defined by UKCRC health category) was less than 1%, only marginally ahead of stroke (including haemorrhagic). By this definition, however, injury and accidents are categorised as fracture, poisoning and burns.

It has been noted by others that funding for injury-related research and overall interest in the development of novel therapeutics in this field lags far behind other public health concerns. Our findings from this scoping study have led us to the same conclusion; that the volume of regenerative medicine research focussed specifically on severe traumatic injury is small, compared to the scale of the problem.

Other literature:

A variety of reviews into regenerative medicine research have been carried out by the UK Government in the last ten years. The following documents were consulted during our scoping study. In addition Surg. Capt. Prof. Rory Rickard sat as a member of the UK Government’s Regenerative Medicine Expert Group (RMEG).

- “Building a viable regenerative medicine industry – a guide to Stakeholders” by *remedi* Management Group, Loughborough University 2010;
- “A bibliometric analysis of Regenerative Medicine” by the Department for Business, Innovation and Skills, 2011;

- “Taking stock of regenerative medicine in the United Kingdom” by Department for Business, Innovation and Skills and Department of Health, 2011
- “A UK Strategy for Regenerative Medicine” by MRC, ESRC, EPSRC, BBSRC and TSB, 2012
- “Stem Cell Report – Trends and perspectives on the evolving international landscape” by Cell Press, 2013
- “Building on our own potential – a UK pathway for regenerative medicine” by the Regenerative Medicine Expert Group (RMEG), 2014
- House of Lords Science and Technology Committee Report on Regenerative Medicine, 1st Report of Session 2013-2014
- “Regenerative Medicine in Europe – global competition, international links and innovation guidance” by the Global Biopolitics Research Group, King’s College, London
- UKRMP First and Second Annual Reports, 2014 and 2015 respectively.
- House of Commons Science and Technology Committee report on Regenerative Medicine, 2017

Requirements capture:

A clinical requirements workshop was held at Dstl in September 2016 attended by clinicians from a variety of specialties within the Defence Medical Services (DMS) to discuss current and future clinical requirements and assess where approaches in regenerative medicine may be applicable. Subsequently, an online survey was developed and sent to all Defence Consultant Advisers and Defence Professors.

External engagement:

In order to further assess the current research landscape dedicated meetings were held, or this review discussed, with a variety of relevant organisations including, but not limited to, those listed below as well as a variety of individual research groups at a wide range of UK academic institutions.

- Department of Health
- UK Regenerative Medicine Platform (UKRMP, a UK Research Council co-funding platform comprising Medical Research Council (MRC), Biotechnology and Biological Sciences Research Council (BBSRC) and Engineering and Physical Sciences Research Council (EPSRC))
- Knowledge Transfer Network (KTN)
- EPSRC Medical Technologies IKC
- NIHR Surgical Reconstruction and Microbiology Research Centre (SRMRC)
- Cell and Gene Therapy Catapult
- NHS Blood and Transplant (NHSBT)
- FortisNet research community
- Centre for Blast Injury Study (CBIS), Imperial College, London
- The Scar Free Foundation

In addition to dedicated meetings and discussions, relevant workshops and conferences were attended, often including presentation of the progress of this scoping study. This gave the opportunity for us to communicate our work and requirements capture and receive valuable feedback.

- Regener8 conference, July 2016

  (Awarded community-sourced session: "Regenerative Medicine at the Frontline" Four oral presentations – A. Spear (Dstl), G. Lawton (RCDM), C. Connon (Newcastle University) and R. Staruch (RCDM))
- Knowledge Transfer Network (KTN) New Collaboration Opportunities in Regenerative Medicine workshop, June 2016
- Characterisation and treatment of wounds workshop, July 2016
- UKRMP inaugural conference, August 2016
- NATO symposium in Regenerative Medicine, October 2016

(Oral presentation – A. Spear (Dstl))

- CBIS (Centre for Blast Injury Studies, Imperial College, London) Regenerative Medicine Networking Event, November 2016
- FortisNet conference, January 2017

  (Oral presentation – A. Spear (Dstl))
- Trauma Innovation conference as part of DSEI, September 2017

  (Oral presentation – A. Spear (Dstl))
- The Scar Free Foundation Symposium, October 2017

International:

As well as the reviews detailed above dealing with international regenerative medicine research, both Dstl and RCDM have access to the research programmes of Allied Nations via initiatives such as The Technical Co-operation Programme (TTCP) and so are cognisant of regenerative medicine research activity in the Defence space of these nations. A review of NATO activity in the regenerative medicine research space was also conducted using open source reports.
